# Colonies for Mental Defectives

**Published:** 1921-08-13

**Authors:** 


### Colonies for Mental Defectives.

At Stoke Park Colony, Stapleton, formerly the Dower House of the family of the Duke of Beaufort, and consisting of about 1,300 acres of farm lands, with the residence, stables, outbuildings, and several more modern structures erected specially, the Hull Corporation receives from 1,500 to 2,000 mental defectives. The land is farmed by the inmates, and all fresh meat, milk, and vegetables consumed by them are produced on the estate. The ages of the patients are from two upwards, and there is one member of the staff to each ten patients, Miss Williamson being the superintendent. The older girls and women are employed principally in housework and gardening. The younger girls are educated on the Montessori system, and attend classes from 9.30 a.m. to noon, when they have dinner, followed by a walk. At 2 p.m. they start school again, and cease work at 4 p.m., when they have recreation. A number of inmates spend their time in spinning and weaving wool grown on the estate. All the fabrics required for their clothing and underclothing are made at the colony, where they are also made up into garments. Mats, rugs, and tapestries of very fine quality are also produced. Leigh Court, Abbots Leigh, is another colony. The house belonged to the late Sir Henry Myers. The principal reception-rooms are used as recreation-rooms. Accommodation for 260 is provided, only high-grade girls from eight to twenty-one being admitted. The land comprises seventy acres, but this is supplemented by the renting of other land. The cowsheds have been converted into excellent schoolrooms. The inmates from eight to sixteen attend school all day. Montessori teaching is again the mode of instruction. Those whose ages are from sixteen to twenty-one spend their time in gardening and housework. A woman gardener is employed, and the only man on the establishment is the park ranger, who also looks after the electric light.

